# Supplementary material for: Confidence in eating disorder knowledge does not predict actual knowledge in collegiate female athletes
Source: PeerJ. 2018 Oct 29;6:e5868. doi: 10.7717/peerj.5868 (PMC6211262; doi:10.7717/peerj.5868)
Supplement: Supplemental Information 3 — Survey that was sent to participants by Google docs survey. [file peerj-06-5868-s004.docx]

# FIVE DOMAINS OF EATING DISORDERS: A SURVEY FOR COLLEGIATE DANCERS (TURK ET AL., 1999)

* Required

Top of Form

1a *

Psychological disturbances are the exclusive cause of eating disorders

- - True
  - False

1b *

Confidence

- - 1-not at all confident
  - 2-not very confident
  - 3-somewhat confident
  - 4- confident
  - 5-very confident

2a *

Females not involved in athletics usually feel more social pressures to be thin than do female athletes

- - True
  - False

2b *

Confidence

- - 1-not at all confident
  - 2-not very confident
  - 3-somewhat confident
  - 4-confident
  - 5-very confident

3a *

Athletes with a negative self image may resort to harmful eating practices in order to improve their performance

- - True
  - False

3b *

Confidence

- - 1-not at all confident
  - 2-not very confident
  - 3-somewhat confident
  - 4-confident
  - 5-very confident

4a *

Coaches can significantly contribute to the development of an eating disorder

- - True
  - False

4b *

Confidence

- - 1-not at all confident
  - 2-not very confident
  - 3-somewhat confident
  - 4-confident
  - 5-very confident

5a *

Individuals with anorexia nervosa usually do not concentrate on academics, however they often strive for excellence in athletics

- - True
  - False

5b *

Confidence

- - 1-not at all confident
  - 2-not very confident
  - 3-somewhat confident
  - 4-confident
  - 5-very confident

6a

Simple dieting is the usual precipitant of an eating disorder

- - True
  - False

6b *

Confidence

- - 1-not at all confident
  - 2-not very confident
  - 3-somewhat confident
  - 4-confident
  - 5-very confident

7a *

Habitual, high intensity exercise in addition to daily team practice may be considered a behavior characteristic of an eating disorder

- - True
  - False

7b *

Confidence

- - 1-not at all confident
  - 2-not very confident
  - 3-somewhat confident
  - 4-confident
  - 5-very confident

8a *

Bulimia nervosa is often termed the "self starvation" syndrome as individuals suffering from bulimia usually ingest less than 500 calories a day

- - True
  - False

8b *

Confidence

- - 1-not at all confident
  - 2-not very confident
  - 3-somewhat confident
  - 4-confident
  - 5-very confident

9a *

It is impossible for an individual who is above average in weight to have an eating disorder

- - True
  - False

9b *

Confidence

- - 1-not at all confident
  - 2-not very confident
  - 3-somewhat confident
  - 4-confident
  - 5-very confident

10a *

The loss of menstration should be considered a potential warning sign of an eating disorder in an athlete

- - True
  - False

10b *

Confidence

- - 1-not at all confident
  - 2-not very confident
  - 3-somewhat confident
  - 4-confident
  - 5-very confident

11a *

Hair loss and/or fine, soft body hair may be seen in those with an eating disorder

- - True
  - False

11b *

Confidence

- - 1-not at all confident
  - 2-not very confident
  - 3-somewhat confident
  - 4-confident
  - 5-very confident

12a *

An obvious decrement in performance will rapidly be seen in an athlete with an eating disorder

- - True
  - False

12b *

Confidence

- - 1-not at all confident
  - 2-not very confident
  - 3-somewhat confident
  - 4-confident
  - 5-very confident

13a *

Due to the psychological considerations involved, only the psychologist and physician should create the treatment plan for an athlete with an eating disorder

- - True
  - False

13b *

Confidence

- - 1-not at all confident
  - 2-not very confident
  - 3-somewhat confident
  - 4-confident
  - 5- very confident

14a *

The team or psychologist should always be the first to approach one suspected of having an eating disorder

- - True
  - False

14b *

Confidence

- - 1-not at all confident
  - 2-not very confident
  - 3-somewhat confident
  - 4-confident
  - 5-very confident

15a *

Once approached about an eating disorder, the athlete will usually be relieved and agree to treatment

- - True
  - False

15b *

Confidence

- - 1-not at all confident
  - 2-not very confident
  - 3-somewhat confident
  - 4-confident
  - 5-very confident

16a *

Treatment of an eating disorder may take years before recovery is complete

- - True
  - False

16b *

Confidence

- - 1-not at all confident
  - 2-not very confident
  - 3-somewhat confident
  - 4-confident
  - 5-very confident

17a *

Athletes diagnosed with an eating disorder should never be allowed to participate in sports until recovery is complete

- - True
  - False

17b *

Confidence

- - 1-not at all confident
  - 2-not very confident
  - 3-somewhat confident
  - 4-confident
  - 5-very confident

18a *

The individual that initially approaches the athlete should concentrate on discussing the eating disorder symptoms identified

- - True
  - False

18b *

Confidence

- - 1-not at all confident
  - 2-not very confident
  - 3-somewhat confident
  - 4-confident
  - 5-very confident

19a *

Distance runners, swimmers, and body builders may have an increased risk for developing an eating disorder

- - True
  - False

19b *

Confidence

- - 1-not at all confident
  - 2-not very confident
  - 3-somewhat confident
  - 4-confident
  - 5-very confident

20a *

Weight monitoring by coaches may increase an athlete's risk for developing and eating disorder

- - True
  - False

20b *

Confidence

- - 1-not at all confident
  - 2-not very confident
  - 3-somewhat confident
  - 4-confident
  - 5-very confident

21a *

A proven, effective means to improve performance is to train at a higher weight then drop or cut weight prior to competition

- - True
  - False

21b *

Confidence

- - 1-not at all confident
  - 2-not very confident
  - 3-somewhat confident
  - 4-confident
  - 5-very confident

22a *

A joking comment regarding an individual's weight may actually trigger an eating disorder in a susceptible athlete

- - True
  - False

22b *

Confidence

- - 1-not at all confident
  - 2-not very confident
  - 3-somewhat confident
  - 4-confident
  - 5-very confident

23a *

Male athletes are not at risk for developing an eating disorder

- - True
  - False

23b *

Confidence

- - 1-not at all confident
  - 2-not very confident
  - 3-somewhat confident
  - 4-confident
  - 5-very confident

24a *

Body fat percentages of 10% or less for females is considered acceptable for athletes that participate in judged sports such as gymnastics, diving or figure-skating

- - True
  - False

24b *

Confidence

- - 1-not at all confident
  - 2-not very confident
  - 3-somewhat confident
  - 4-confident
  - 5-very confident

25a *

Educating coaches about eating disorders is an important preventative strategy for minimizing the risk of such disorders

- - True
  - False

25b *

Confidence

- - 1-not at all confident
  - 2-not very confident
  - 3-somewhat confident
  - 4-confident
  - 5-very confident

26a *

If the athletic department does not offer education programs about eating disorders, then it is the coach's responsibility to educate him/herself on the topic

- - True
  - False

26b *

Confidence

- - 1-not at all confident
  - 2-not very confident
  - 3-somewhat confident
  - 4-confident
  - 5-very confident

27a *

Proper nutrition, body composition, and weight maintenance are issues that should be included in educational programs for athletes.

- - True
  - False

27b *

Confidence

- - 1-not at all confident
  - 2-not very confident
  - 3-somewhat confident
  - 4-confident
  - 5-very confident

28a *

The medical staff should be the only individuals to conduct weigh-ins for athletes

- - True
  - False

28b *

Confidence

- - 1-not at all confident
  - 2-not very confident
  - 3-somewhat confident
  - 4-confident
  - 5-very confident

29a *

In order to improve individual performance, coaches should emphasize the importance of achieving an ideal body weight and body composition, in addition to proper training

- - True
  - False

29b *

Confidence

- - 1-not at all confident
  - 2-not very confident
  - 3-somewhat confident
  - 4-confident
  - 5-very confident

30a *

Dieting may be encouraged by coaches in cases where weight loss is needed in a short time period

- - True
  - False

30b *

Confidence

- - 1-not at all confident
  - 2-not very confident
  - 3-somewhat confident
  - 4-confident
  - 5-very confident

Demographics *

*note: Height and weight were manually inserted in an open response box

Height

Bottom of Form

Demographics *

Weight (pounds)

Demographics *

Age

- 17
- 18
- 19
- 20
- 21
- 22
- 23
- 24+

Demographics *

What Sport do you participate in for your college? (If you play more than one sport, please use your more preferred sport)

- Cross country/Distance running
- Track-Sprints/Jumps
- Track-Throws

*note: responses labeled as “other” were replaced with basketball as authors realized basketball was the only varsity sport not listed that Baker athletes could participate (See comment below)

- Soccer
- Golf
- Volleyball
- Cheer/Dance
- Softball
- Tennis
- Other

Demographics *

Which University do you attend?

*note: the original design was to survey athletes at multiple institutions, but due to low numbers, the university grouping variable was dropped and only 51 responses from Baker University were recorded.

- Baker University
- Avila University
- Ottawa University
- Mid America Nazarene University
- Benedictine College
- Haskell Indian Nations University
- Park University

| **?** | **Answers** |
| --- | --- |
| **1** | **F** |
| **2** | **F** |
| **3** | **T** |
| **4** | **T** |
| **5** | **F** |
| **6** | **T** |
| **7** | **T** |
| **8** | **F** |
| **9** | **T** |
| **10** | **T** |
| **11** | **T** |
| **12** | **F** |
| **13** | **F** |
| **14** | **F** |
| **15** | **F** |
| **16** | **T** |
| **17** | **F** |
| **18** | **F** |
| **19** | **T** |
| **20** | **T** |
| **21** | **F** |
| **22** | **T** |
| **23** | **F** |
| **24** | **F** |
| **25** | **T** |
| **26** | **T** |
| **27** | **T** |
| **28** | **T** |
| **29** | **F** |
| **30** | **F** |
