# Supplementary figures and images for: Confidence in eating disorder knowledge does not predict actual knowledge in collegiate female athletes
Source: PeerJ. 2018 Oct 29;6:e5868. doi: 10.7717/peerj.5868 (PMC6211262; doi:10.7717/peerj.5868)

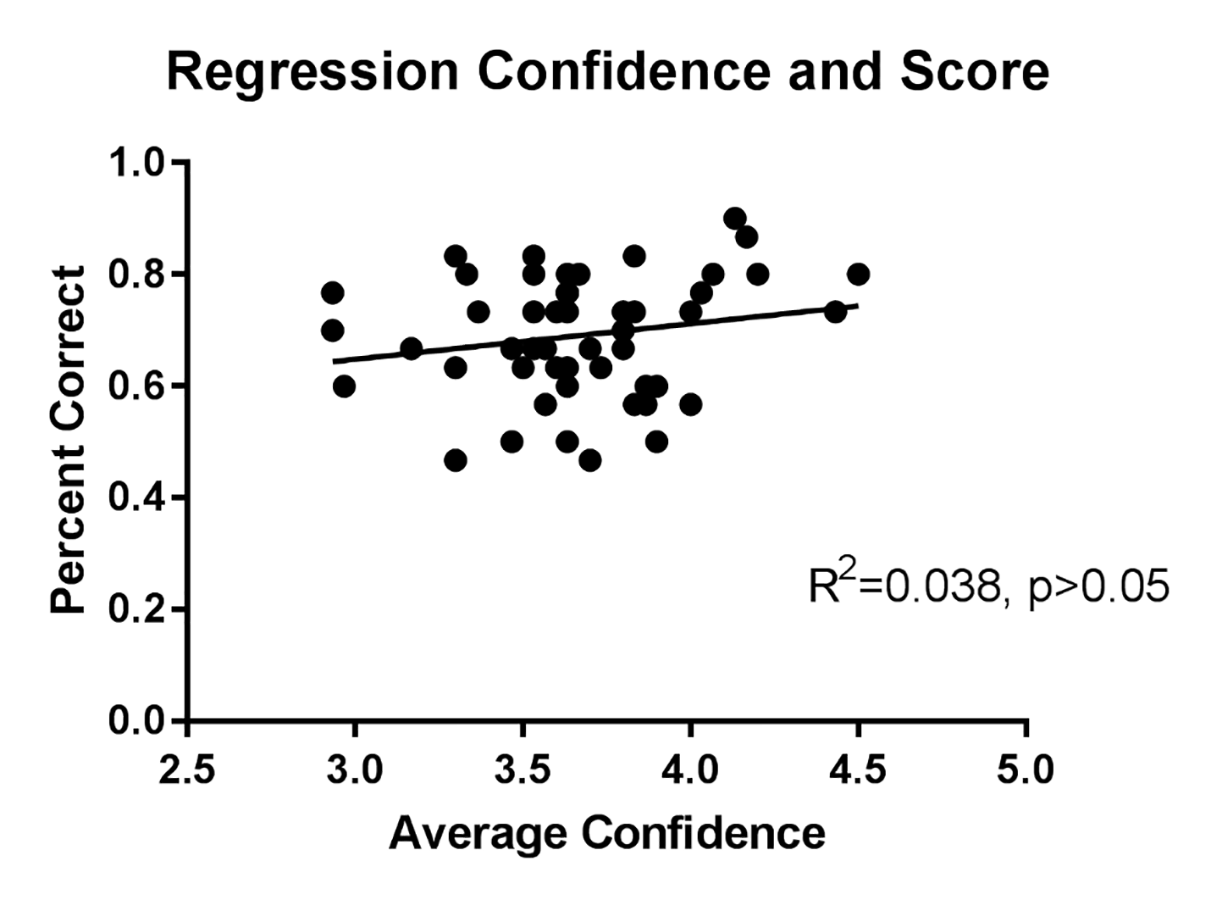

Supplement: Figure S1 — Regression of average confidence on total scores. Average confidence was calculated by summing all confidence scores then dividing by total possible confidence (corresponding to a confidence score of 5 for each question). We noted no sigfnicant relationship between confidence and total score (F(1, 49) = 1.94, R2 = 0.038, b = 0.063, p > 0.05). [file peerj-06-5868-s005.png]

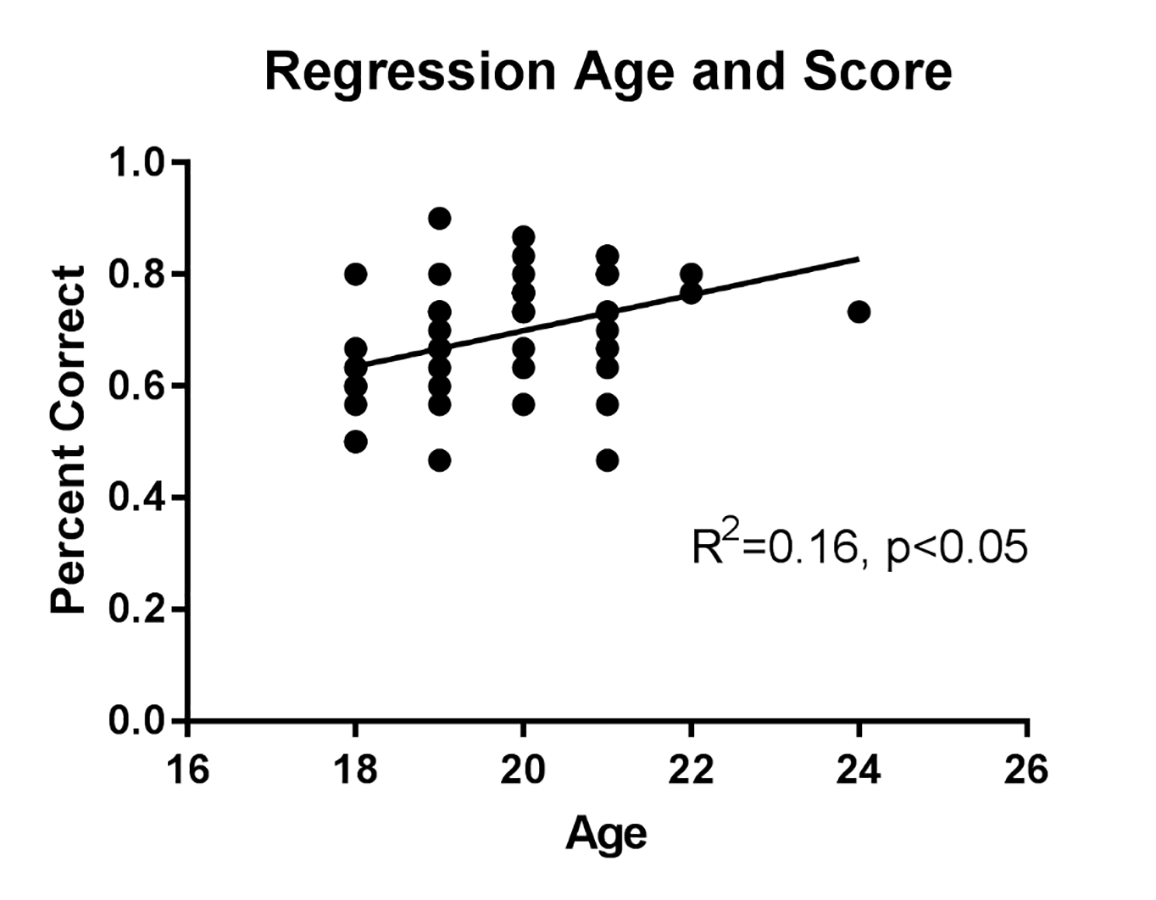

Supplement: Figure S2 — Regression of age on total scores, we noted a small significant relationship between age and total score (F(1, 49) = 9.17, R2 = 0.16, b = .0321, p < 0.05). [file peerj-06-5868-s006.png]
